# Antitumor Activities of Aqueous Cinnamon Extract on 5637 Cell Line of Bladder Cancer through Glycolytic Pathway

**DOI:** 10.1155/2022/3855368

**Published:** 2022-08-11

**Authors:** Zeynab Aminzadeh, Nasrin Ziamajidi, Roghayeh Abbasalipourkabir, Sajedeh Daei, Sobhan Helbi, Abbas Moridnia

**Affiliations:** ^1^Department of Clinical Biochemistry, School of Medicine, Hamadan University of Medical Sciences, Hamadan, Iran; ^2^Molecular Medicine Research Center, Hamadan University of Medical Sciences, Hamadan, Iran; ^3^School of Medicine, Dezful University of Medical Sciences, Dezful, Iran; ^4^Department of Genetic and Molecular Biology, School of Medicine, Dezful University of Medical Sciences, Dezful, Iran

## Abstract

**Background:**

Pharmacotherapy with medicinal plants is a promising approach to treat cancer. Cinnamon is a medicinal plant whose properties have been proven in various fields of medical sciences. Among its biological activities, its antioxidant and antiviral effects can be mentioned. In this study, the antitumor effects of Cinnamon with a focus on glucose metabolism in bladder cancer carcinoma cell-line 5637 were investigated.

**Methods:**

Aqueous extract of Cinnamon was prepared from Cinnamon bark. Bladder cancer 5637cell line were treated with different concentrations of aqueous extract of Cinnamon. MTT was used to evaluate cell viability at 24, 48, and 72 h. The concentration of 1.25, 2.50, and 5 mg/ml was used. Apoptosis was assessed with Hochest33258 staining. For evaluating of aqueous extract of Cinnamon effect on glycolysis, the gene expression of epidermal growth factor receptor 2 (ErbB2), heat shock protein transcription factor1 (HSF1), and lactate dehydrogenase *A* (LDHA), as well as protein levels of HSF1 and LDHA, LDH activity, glucose consumption, and lactate production, were measured.

**Results:**

Aqueous extract of Cinnamon significantly decreased ErbB2, HSF1, and LDHA gene expression and also decreased the protein level of HSF1 and LDHA, LDH activity, glucose consumption, and lactate production dose-dependently (*p* < 0.05).

**Conclusion:**

Our finding showed that the aqueous extract of Cinnamon can inhibit proliferation in 5637 cells by inhibition of glycolysis and induction of apoptosis.

## 1. Introduction

Bladder cancer is the 10th most common cancer worldwide, according to the World Health Organization. The rapid and progressive spread of this cancer has become a major concern in the world and kills millions of people every year [1]. An unhealthy lifestyle, alcohol, tobacco use, and other factors can play an important role in the development of many cancers and chronic diseases such as diabetes and cancer. Environment, nutritional, and occupational factors can activate oxidative stress in the body [2–4].

Oxidative stress is a stimulus for the onset and development of cancer because this process begins with the activation of inflammatory processes in the body and eventually ends with the development of chronic diseases such as diabetes, cardiovascular disease, cancer, and many disorders [5]. Second, oxidative stress helps convert normal cells to cancer cells, the progression, migration, recurrence of cancer, and many other features that are beneficial for cancer cells. Oxidative stress also helps convert normal cells to cancer cells, the progression, migration, recurrence of cancer, and many other features that are beneficial for cancer cells [5–7].

At first, the body's response to induced oxidative stress is mediated by a defense mechanism called the thermal shock response which is regulated by a transcription factor called heat shock protein transcription factor1 (HSF1). HSF1 is known as a powerful factor with different functions, that in addition to its role in the defense responses is also involved in regulating many pathways related to cell growth, proliferation, and metabolism such as glycolysis and lipid metabolism [8, 9]. In addition, various studies revealed that HSF1 is associated with metastasis, cancer cell survival, and tumor proliferation. In other words, elevated expression of HSF1 has been observed with a poor prognosis of cancer [10]. This association is due to the role of HSF1 in activating pathways such as protein kinase C (PKC) [11], nuclear factor-kappaB (NF-*κ*B) [12], phosphoinositide 3-kinase (PI3K), Akt/mammalian (or mechanistic) target of rapamycin (mTOR) [8], which are essential for proliferation, resistance to apoptosis, migration, invasion, and cancer cell metastasis [8, 10, 13, 14].

Epidermal growth factor receptor 2 (ErbB2) is a potent oncogene associated with many cancers, including breast cancer, and its increased expression has been seen in the poor prognosis of cancer. This oncogene is a regulator of cell bioenergetic pathways such as ras, PI3K/AKT, and mTOR [15–17]. Inhibition of ErbB2 can reduce the expression of HSF1 [18].

Unlike normal cells, the energy supply in cancer cells is mainly through the anaerobic pathway or glycolysis [19]. Continuation of glycolysis in cancer cells requires the supply of NADH, produced by lactate dehydrogenase (LDH). One of that isoenzymes is LDHA, which can be increased in most cancers [20]. Increased HSF1 can act a rule as a transcription factor and cause evaluate the expression of lactate dehydrogenase A (LDHA), and oppositely reducing HSF1 can decrease the expression of LDHA, which targets one of the vital processes of cancer cells, which is actually glycolysis [21]. Therefore, due to the importance of glycolysis for cancer cells' survival, targeting these factors as regulators for glycolysis can be a good target for cancer treatment [22].

Since chemotherapy drugs have many side effects, researchers have always been looking for natural compounds that are the least harmful to the body. The Cinnamon plant, known as Cinnamon and Cinnamomum, belongs to the Lauraceae family and has been used as a spice in many countries for centuries. In addition to its use in cooking, it has been used as a gastrointestinal pain reliever in some diseases [23]. Cinnamon has various medicinal properties such as antioxidant [24, 25], anti-viral [26–28], anti-inflammatory [29], and anti-diabetic [30] effects.

In this study, we investigated the effects of the aqueous extract of Cinnamon on human bladder carcinoma cell line 5637, especially on the gene expression of HSF1, ErbB2, and LDHA and the level of LDHA and HSF1 proteins and apoptosis.

## 2. Material and Method

### 2.1. Preparation of Aqueous Extract of Cinnamon

Cinnamon sticks were purchased from a local market in this study. *Cinnamomum cassia* confirmed by an herbalist was used. It was then grounded. 250 g of the powder was taken and soaked in 2500 ml of water for 24 h at room temperature. Then it was boiled at 100°C for 30 min. Afterward, it was filtered and the resulting solution was lyophilized and stored at −20°C until used [31].

### 2.2. HPLC Analysis

HPLC analysis was performed to determine the amount of Cinnamaldehyde in the prepared extract. Commercial Cinnamaldehyde manufactured by Sigma (Cat. No. W228613) is used as standards makers for the quality control of the composition of Cinnamaldehyde in the aqueous extract of Cinnamon in each experiment. Chromatography was performed by 1% acetic acid (H20)-MeOH (50 : 50 v/v) at room temperature on a Phenomenex Luna 5u C18, 100 A pore size, 250 × 4.60 mm I.D. column. The flow rate of the mobile phase was 1 ml/min [32]. According to the results, the amount of Cinnamaldehyde in the prepared extract was 26 *μ*g/ml.

### 2.3. Cell Culture

Human bladder carcinoma cell line 5637 was purchased from Pasteur Institute, Tehran, Iran. The cells were cultured in RPMI1640 (Gibco RL, Grand Island, NY) medium with 10% FBS (Gibco RL, Grand Island, NY), 20 mM HEPES, 2 mM glutamine, and 1% of penicillin-streptomycin in 37°C incubators under 5% CO_2_. When the confluency of cells reached 85%, experiments were performed.

### 2.4. MTT Colorimetric Assay

Cell viability was evaluated by the MTT method to determine the optimal concentration and time for treatment of cells [33] with aqueous extract of Cinnamon. 2 × 10^4^ cells were seeded in 96 well plates. The cells incubated with concentrations of 0.16, 0.32, 0.64, 1.25, 2.50, 5, 10, and 20 mg/ml aqueous extract of Cinnamon at 37°C under 5% CO2 for 24, 48, and 72 h [34]. After treatment, 20 *μ*l of MTT working solution was added to each well and incubated at 37° for 3 h. The supernatant was collected and the formazan crystals were dissolved in DMSO (100 *μ*l/sample). Finally, cell viability was assessed by measuring light absorption at 590 nm by using a plate reader (Biotech Instruments Inc., Winooski, USA).

### 2.5. Hochest33258 Staining

The Hochest staining 33285 (Sigma, USA) was used to evaluate the occurrence of apoptosis. First, the cells were cultured in plates of 6 wells (10^6^ cells/well). After 24 h treatment, the cells were trypsinized and the contents of each well were transferred to a separate microtube. Then, the tubes were centrifuged at 1500*g* for 5 min. After removing the supernatant, 100–200 *μ*l of absolute cold methanol was added to the precipitate for cell fixation and incubated for 15 min at −20°C. After incubation, centrifuged again and added 50 *μ*l PBS and 1 *μ*l ready-to-use Hochest solution (1 *μ*l Hochest stock per 100 *μ*l PBS buffers) to the cell precipitate and incubate for 30 minutes at laboratory temperature. It was centrifuged again and 100 *μ*l PBS was added to the precipitate. Then mixed and transferred to a slide for observation using a fluorescence microscope.

### 2.6. Total RNA Extraction and Real-Time PCR

Total RNA was extracted by KIAZOL (KIAZIST, Iran) after treating the cells with specific concentrations of aqueous extract of Cinnamon. The quantity and quality of the extracted RNA were determined by NanoDrop ND-1000 spectrophotometer and 1% agarose gel electrophoresis, respectively. Synthesis of cDNA from total RNA was performed with a cDNA synthesis kit (Biofact, Korea) according to the manufacturer's instructions. Polymerase chain reaction (PCR) was performed using gene-specific primers and SYBR Green kit (Biofact, Korea). Following primers were used; Glyceraldehyde 3-phosphate dehydrogenase(GAPDH) 5′-AAGGCTGTGGGCAAGGTCATC-3′ (forward) and 5′-GCGTCAAAGGTGGAGGAGTGG-3′ (reverse), HSF1: 5′-GAAGGGGAAGCAGGAGTG-3′ (forward) and 5′-GTTGACGACTTTCTGTTGCTG-3′ (reverse), ErbB2: 5′-TGATAGACACCAACCGCTCT-3′ (forward) and 5′-CAGAACTCTCTCCCCAGCA-3′ (reverse) and LDHA: 5′-GGTCCTTGGGGAACATGGAG-3′ (forward) and 5′-TAGCCCAGGATGTGTAGCCT-3′ (reverse). GAPDH is housekeeping gene and here it was used as internal control. The specificity of PCR products was verified by gel electrophoresis in 1% agarose. Finally, the relative genes expression was evaluated by 2^−ΔΔCt^ method.

### 2.7. Western Blotting

A total of 5637 cells were treated and then lysed in RIPA buffer (KIAZIST, Hamadan, Iran) supplemented with protease inhibitor cocktail (KIAZIST, Iran). Then agitated for 30 min at 4°C. Next, centrifuged (10 min 12,000*g* at 4°C), the supernatant was collected and protein concentration was assayed by the bicinchoninic acid (BCA) method (BCA, Thermo Scientific pierce, USA). Protein (40 *μ*g) was loaded on 10% SDS-polyacrylamide gels and then transferred to the nitrocellulose membrane. The membrane was blocked with 5% non-fat dried milk in TBS-T (50 mM Tris-HCl, pH 7.6, 100 Mm NaCl, and 0.1% Tween20) for 1 h. Subsequently, the membrane was incubated with anti-HSF1(1 : 2000 dilution), anti-LDHA (1 : 1000 dilution) and anti-*β*actin (1 : 2000 dilution) antibodies overnight at 4°C. For visualizing the immunoreactivity of proteins enhanced chemiluminescence (ECL) was used. Finally, the band intensity was quantified using ImageJ software (NIH, Bethesda, USA). *β*-actin was used as the internal control to normalize intensity values.

### 2.8. LDH Activity Assay

Treated cells were lysed by freeze-thawing and then used PBS buffer supplemented with antiprotease. afterward, it was centrifuged at 15000*g* for 15 min. The obtained lysates were used to measure activity LDH according to the manufacturers' instructions kit (Pars Azmun, Iran). Total protein was also quantified by bicinchoninic acid assay (BCA, Thermo Scientific Pierce, USA).

### 2.9. Measurement of the Glucose Consumption and Lactate Production

Glucose intake and lactate production was measured in the supernatant of media after treatment. The cells were treated with certain concentrations of aqueous extract of Cinnamon for 24 h, then the supernatant was collected and used to evaluate lactate and glucose according to the kit manufacturer's instructions (Pars Azmun, Iran).

### 2.10. Statistical Analysis

Data analysis was performed by IBM SPSS Statistics 16.0 (IBM, USA) and Microsoft Graph Pad Prism 6 (Graph Pad Prism Inc., USA) used for drawing graphs. To determine statistical differences, we performed one-way ANOVA and Tukey's post-test. Results were represented as mean ± standard division (SD). *P* < 0.05 was considered statistically significant.

## 3. Result

### 3.1. MTT Assay and Cell Viability

**Figure 1:**
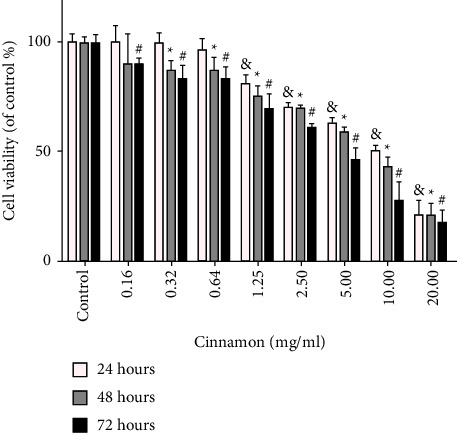
Effect of different concentrations of Cinnamon aqueous extract on 5637cell viability. 5637cell were treated with concentrations (0.16, 0.32, 0.64, 1.25, 2.50, 5, 10, and 20 mg/ml) for 24, 48, and 72 h Results are expressed as mean ± SD and each value is the average of at least three independent replicates. & denoted the significant difference in comparison with the control group in 24 h (*P* < 0.05), ^*∗*^ in 48 h (*P* < 0.05), ^#^ in 72 h. (*P* < 0.05) compared to the control group.

The results obtained from the MTT assay showed that aqueous extract of Cinnamon has time and dose-dependent effects on 5637 cells, as shown in Figure 1. After 24, 48, and 72 h treatment of 5637 cells with (0.16, 0.32, 0.64, 1.25, 2.50, 5, 10, and 20 mg/ml) aqueous extract of Cinnamon concentrations MTT test was performed. At 72 h, concentrations above 2.50 mg/ml were toxic to cells. At 48 hours after treatment, concentrations above 5 mg/ml showed cell viability below 50% but cell viability at 24 h, for all used concentrations was above 50%, except 10 and 20 mg/ml. According to the results obtained from MTT, the IC50 was calculated at 10 mg/ml. Therefore, concentrations of 1.25, 2.50, and 5 mg/ml and 24 h were selected to continue the experiments.

### 3.2. Apoptosis Assay and Hochest33258 Staining

**Figure 2:**
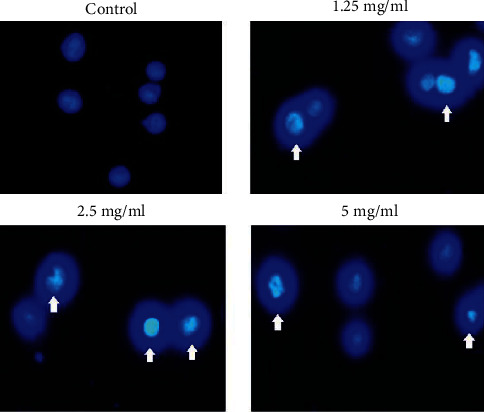
Effect of different concentrations of Cinnamon aqueous extract on 5637cell apoptosis by Hochest33258 staining. All ×40 magnification images are obtained by fluorescence microscopy.

The Hochest staining is used for investigating apoptosis in cell culture. In this method, normal cells are seen uniformly and have a normal nucleus, whereas apoptotic cells' nuclei have dense Chromatin and nuclear fragmentation and seem irregular appearance, also they seem as bright spots. For this purpose, 5637 cells were treated for 24 h with 1.25, 2.50, and 5 mg/ml aqueous extract of Cinnamon. Then cells were stained with Hochest33258 followed by observation under a fluorescence microscope. As shown in Figure 2, bright spots that represented apoptosis in cells and irregular luminosity compared to control were seen. Therefore, it can be concluded that aqueous extracts of Cinnamon induce apoptosis at these concentrations.

### 3.3. mRNA Expression of HSF1, ErbB2, and LDHA in 5637 Cells

**Figure 3:**
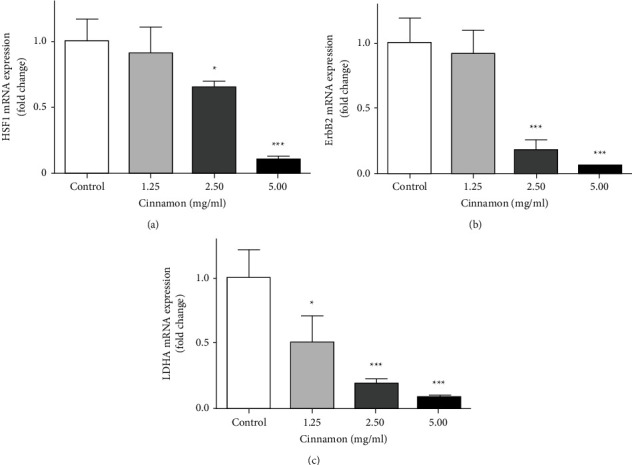
Effect of different concentrations of Cinnamon aqueous extract on mRNA expression (fold change) of HSF1 (a), ErbB2 (b), and LDHA (c). Data are represented as the mean ± SD. Each value is the average of at least three independent replicates. (^*∗*^*P* < 0.05, ^*∗∗∗*^*P* < 0.001compared to the control group). ErbB2: human epidermal growth factor receptor2, HSF1: heat-shock transcription factor1, LDHA: lactate dehydrogenase A.

To determine the effect of the aqueous extract of Cinnamon on the inhibition of glucose metabolism in 5637 cells, the expression of genes HSF1, ErbB2 and LDHA were evaluated. Our results showed that different aqueous extracts of Cinnamon in selected concentrations could reduce the expression of all three genes in a dose-dependent condition compared to the control group. As shown in Figure 3(a), gene expression of HSF1 was significantly reduced at concentrations of 2.50 (*P* < 0.05) and 5 mg/ml (*P* < 0.001). At a concentration of 1.25 mg/ml, the decrease was not significant compared to the control group (*P* > 0.05). Aqueous extract of Cinnamon reduced, significantly the expression of ErbB2 for 2.50 and 5 mg/ml (*P* < 0.001). There was no significant change for 1.25 mg/ml compared to the control group (*P* > 0.05) (Figure 3(b)). LDHA gene expression was significantly lowered in all three concentrations (Figure 3(c)), thus there were significant changes in concentrations of 1.25 mg/ml (*P* < 0.05), 2.50, and 5 mg/ml (*P* < 0.001).

### 3.4. Level of HSF1 and LDHA Proteins in 5637 Cells

**Figure 4:**
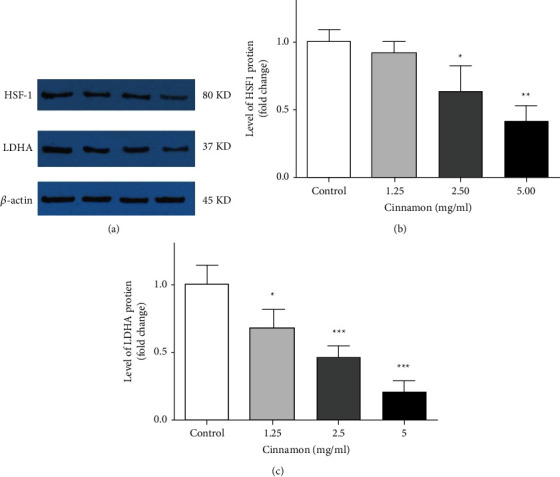
Effect of different concentrations of Cinnamon aqueous extract on 5637cell. (a) The image of protein extraction by the immunoblotting result, (b) HSF1, and (c) LDHA protein expression. Data are reported as the mean ± SD and each value is the average of at least three independent replicates. (^*∗*^*P* < 0.05, ^*∗∗*^*P* < 0.01 and ^*∗∗∗*^*P* < 0.001 compared to the control group). HSF1: heat-shock transcription factor1, LDHA: lactate dehydrogenase A.

The results showed that level of HSF1 and LDHA protein in treated cells with aqueous extract of Cinnamon was significantly reduced. As shown in Figure 4(b), the level of HSF1 in 2.50 (*P* < 0.05) and 5 mg/ml (*P* < 0.01) was significantly decreased. The concentration of 1.25 mg/ml showed no significant change compared to the control group (*P* > 0.05). LDHA protein expression was significantly reduced in three used concentrations. This diminution in protein levels was seen as dose-dependent significantly compared to lower concentrations (Figure 4(c)), which was for 1.25 mg/ml (*P* < 0.05), 2.50 mg/ml, and 5 mg/ml (*P* < 0.001).

### 3.5. LDH Activity

**Figure 5:**
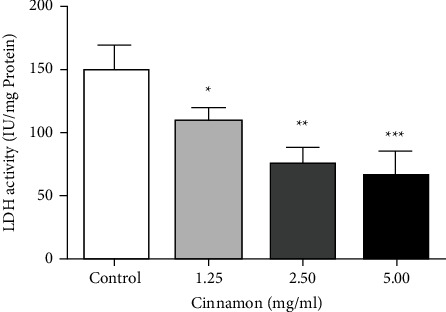
Effect of different concentrations of Cinnamon aqueous extract on LDH activity in5637cells. Data are reported as the mean ± SD. All reported value is the average of at least three independent replicates. (^*∗*^*P* < 0.05, ^*∗∗*^*P* < 0.01 and ^*∗∗∗*^*P* < 0.001 compared to control group). LDH: lactate dehydrogenase.

To confirm the effects of the aqueous extract of Cinnamon on the reduction of glycolysis, an LDH activity assay was performed. According to Figure 5, aqueous extract of Cinnamon could reduce LDH activity. LDH activity was significantly reduced at 1.25 (*P* < 0.05), 2.5 (*P* < 0.01), and 5 mg/ml (*P* < 0.001) compared to the control group.

### 3.6. Glucose Consumption and Lactate Production

**Figure 6:**
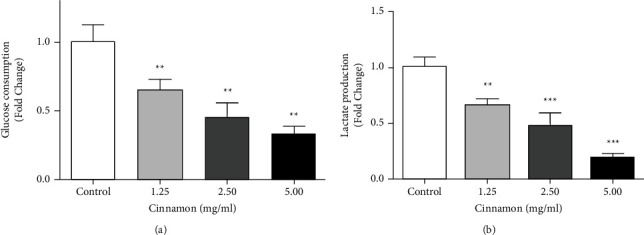
Effect of different concentrations of Cinnamon aqueous extract on glucose consumption (a) and lactate production (b) in 5637 cells. Data are represented as the mean ± SD. All value is the average of at least three independent replicates. (^*∗∗*^*P* < 0.01 and ^*∗∗∗*^*P* < 0.001 compared to the control group).

Glucose uptake and lactate production in media was assayed to ensure glycolysis inhibition. Results were shown (Figure 6) that glucose consumption and lactate production were significantly decreased in three used concentrations time and dose-dependently. Glucose intake for 5637 cells from media after 24 h treatment was significantly decreased in all used concentrations (*P* < 0.01) (Figure 6(a)). Analysis of data obtained from lactate consumption by 5637 cells in concentrations of 1.25 (*P* < 0.01), 2.5 and 5 mg/ml (*P* < 0.001) showed a significant decrease in lactate consumption compared to the control group (Figure 6(b)).

## 4. Discussion

The World Health Organization estimates cancer as the first or second leading cause of death before the age of 70 in most countries. This issue caused finding the best way for controlling or treating cancer be the priority for researchers who work in this field. Although chemotherapy is routinely used to treat many cancers, sometimes effective yet mostly has hazardous side effects that are limiting its therapeutic potential [35–37].

The use of plants such as Cinnamon has always been considered in medical sciences due to their proven properties. Various compounds in the Cinnamon plant, such as Cinnamaldehyde, have made it possible to be effective in cancer therapy or chemoprevention by various mechanisms such as inhibition of NF-KB or activated protien1 (AP1) [32], angiogenesis [38], and vascular endothelial growth factor (VEGF) pathways [39]. In this study, we showed that Cinnamon reduced the expression of ErbB2, HSF1, and LDHA at transcriptional and translational levels. In the present study, it was seen that cinnamon extract reduced all three ErbB2, HSF1, and LDHA genes. The relationship between these three genes has been proven in a study conducted in 2009 by Zhao et al. They observed that with the decrease in the expression of ErbB2, the expression level of LDHA gene and enzyme activity of LDH both decrease. They also reported that when siRNA was used to inhibit LDHA gene expression, in addition to inhibiting glucose uptake into cell cytoplasm and lactate production, it can reduce cell growth and prevent overexpression of ErbB2. They also observed that the amount of LDHA also decreased following a decrease in HSF1 in the cell, and this could be due to the direct effect of the expression of HSF1 as a transcription factor [21]. Different mechanisms are related to the ErbB2-HSF1-LDHA pathway. Overexpression of ErbB2 was seen in bladder cancer and is associated with poor prognosis [40–42]. ErbB2 calls PI3K and then AKT and mTOR through the signaling cascade it activates. HSF1 is an activation target for mTOR that activates HSF1 by phosphorylation of serine 326. By activating HSF1, proteins called HSPs are activated. Each of the HSPs serves the cancer cell in certain ways. HSPs proteins participate in many processes such as angiogenesis, metastasis, and cancer cell metabolism [43, 44]. HSP27,70 and 90 have an important role in the reactivation of ErbB2, which reactivates the entire mTOR pathway as an important way for cancer cell survival. HSP90 expression is directly affected by HSF1. HSP90 stabilizes many proteins such as platelet-derived growth factor receptor (PDGFR), vascular endothelial growth factor receptor (VEGFR), and even ErbB2 [10]. Ras, raf, ERK, and the MAPK pathway are all activated by ErbB2. All of these events occur in the form of direct feedback, followed by activation one after the other. The result of these events is an increase in transcription from intracellular glucose transporter genes and other genes that are involved in energy production, such as lactate dehydrogenase, phosphofructokinase, and pyruvate kinase, which is further evidence of altered metabolism in cancer cells known as the Warburg effect [45]. On the other hand, HSF1 plays an important role as a transcription factor in regulating a process called metabolic reprogramming in cancer cells. HSF1 also directly increases its expression by acting on the LDHA gene. The pyruvate dehydrogenase kinase 3 gene is another target for HSF1, which inhibits the conversion of glucose to Acetyl CoA by inactivating the pyruvate dehydrogenase complex, thus ensuring the continuation of glycolysis in cancer cells [46]. On the other hand, HSF1 recruits methyltransferase DNMT3 to the MN317 lncRNA gene region. These methyltransferases prevent the activation of MIR317 by hypermethylating this gene region. Because MIR317 causes the activation of glutaminase GLS1, which helps to reactivate mTOR by hydrolyzing glutamine. Thus, HSF1, which is a target for mTOR, is also reactivated [10, 47, 48] for the first time Current study showed an aqueous extract of Cinnamon, is able to reduce the expression of ErbB2 and HSF1; consequently, the expression of LDHA is also reduced. Also, we measured LDH activity, glucose uptake, and lactate production, which indicate glycolysis and lactate dehydrogenase activity, respectively. Results confirmed an aqueous extract of Cinnamon can reduce glucose metabolism in the 5637 cell line.

Moreover, we are using Hochest23258 to detect apoptotic cells and four finding shows Cinnamon could induce apoptosis in 5637 cells, which is consistent with previous studies. Soumya et al. showed that in cervical cancer cells aqueous extract of Cinnamon increases intracellular calcium signaling and alters the permeability of mitochondrial potential consequently inducing apoptosis [49]. Gwang et al. demonstrated water extract of Cinnamon can induce apoptosis in a colorectal cancer cell by ROS-dependent NF-*κ*B and ATF3 activation [50]. Another point is the importance of HSF1, it has a vital role in protecting cancer cells from apoptosis and helping them to invade from the immune system by metastasis. Therefore, decreased expression and inactivation of HSF1, has protecting effect on the host [51, 52]. In fact, Cinnamon and its active components kill cancer cells directly by their regulating act on transcription factors such as HSF1, and kill indirectly by increasing of ROS production and mitochondrial dysfunction. Cinnamaldehyde is the active ingredient in Cinnamon extract, which can induce apoptosis in different cancer cells. In this regard, we can refer to the study of Hyeon et al. in 2003. It is one of the first published reports about the inhibitory properties of Cinnamaldehyde in cancer cells in HL-60 cells. They showed Cinnamaldehyde can impair the cell's oxidative system by increasing ROS production and subsequently induce apoptosis by increasing mitochondrial membrane permeability and cytochrome C releasing [53]. In the next years after 2003, a lot of research was done about the inhibitory effect of Cinnamaldehyde and Cinnamon extract, they confirmed the report of Hyeon et al with different mechanisms. In 2020, Lee et al. showed that Cinnamaldehyde induces cell apoptosis by interfering with mitochondrial function, disrupting mitochondrial membrane balance, and increasing permeability (∆M*ψ*), as well as increasing intracellular calcium [54].

## 5. Conclusion

Our results suggest that the aqueous extract of Cinnamon suppresses 5637 cells proliferation through inhibition of glycolysis, and induces apoptosis. From these findings, the aqueous extract of Cinnamon can act as a natural candidate for the development of chemoprevention or therapeutic agents in human bladder cancer.

## Data Availability

All data have sufficient accuracy and precision. All the reported results are obtained from the detailed analysis of the raw data obtained from the experiments.
